# Peripheral Galanin Receptor 2 as a Target for the Modulation of Pain

**DOI:** 10.1155/2012/545386

**Published:** 2012-01-24

**Authors:** Richard P. Hulse, Lucy F. Donaldson, David Wynick

**Affiliations:** ^1^School of Physiology and Pharmacology, University of Bristol, University Walk, Bristol BS8 1TD, UK; ^2^School of Clinical Sciences South Bristol, University of Bristol, University Walk, Bristol BS8 1TD, UK

## Abstract

The neuropeptide galanin is widely expressed in the nervous system and has an important role in nociception. It has been shown that galanin can facilitate and inhibit nociception in a dose-dependent manner, principally through the central nervous system, with enhanced antinociceptive actions after nerve injury. However, following nerve injury, expression of galanin within the peripheral nervous system is dramatically increased up to 120-fold. Despite this striking increase in the peripheral nervous system, few studies have investigated the role that galanin plays in modulating nociception at the primary afferent nociceptor. Here, we summarise the recent work supporting the role of peripherally expressed galanin with particular reference to the dual actions of the galanin receptor 2 in neuropathic pain highlighting this as a potential target analgesic.

## 1. Introduction

The 29-amino-acid neuropeptide galanin was first identified in porcine intestine [1] and later in the rat central nervous system and intestine [2]. Since then galanin has been shown to play important roles in a number of physiological processes including cognition [3], feeding [4], and nociception [5]. This paper will consider activation of galanin receptors on primary afferent nociceptors as a possible target for pain treatment.

## 2. Galanin-Historical Perspectives and Spinal Nociceptive Processing

Galanin is expressed in many areas of the nervous system involved in somatosensation including the dorsal root ganglia (DRG) and spinal cord [6, 7], and also in other CNS regions such as the arcuate nucleus and periaqueductal grey [8, 9]. In the peripheral nervous system, low levels of galanin expression is present in the DRG of intact adult rodents, with the peptide expressed in fewer than 5% of DRG sensory neurons [10]. These galanin-expressing neurons belong to a group of small diameter sensory afferents that respond to capsaicin [7, 11], which are characteristically C fibre nociceptors [12]. Galanin is now considered to be an injury-response peptide, as it is dramatically upregulated in DRG neurons in sciatic [6, 10] and saphenous nerve injury models [13–17]. The original observations of galanin upregulation after peripheral nerve injury strongly suggested a functional role for galanin in nociception and that these actions were through modulation of spinal nociceptive processing.

Prior to the identification and characterization of galanin receptors in the central nervous system, functional studies demonstrated that galanin could modulate spinal nociceptive reflexes. Behaviourally, intrathecal galanin administration was initially reported to have differential effects on thermal and mechanical nociception in the normal animal; thermal responses were inhibited whereas mechanical responses were enhanced [18, 19]. Numerous further studies resulted in the recognition that galanin has differential actions on spinal nociceptive processing, in that low galanin concentrations exert pronociceptive [20–23] and higher concentrations lead to antinociceptive effects [24, 25]. In contrast, in nerve-injured rodents, intrathecal galanin has predominantly antinociceptive actions at the spinal level, acting on a greater number of neurons, and these actions are more pronounced than those seen in naïve animals [26–32].

These apparently conflicting spinal actions of galanin at different concentrations are thought to be due to the differential distributions, and/or activation of the galanin receptor subtypes. To date, three galanin G-protein-coupled receptor subtypes have been identified, galanin receptor (GalR) 1 [33], GalR2 [34], and GalR3 [35]. Galanin binding is abundant in the superficial laminae of the dorsal horn, being localized to GABA and glycine containing inhibitory interneurons [36]. The lack of specific antibodies against the galanin receptors [37] has hampered localisation attempts, but GalR1 mRNA is abundant in the superficial laminae of the dorsal horn [38, 39], in glutamatergic neurons [40]. GalR2 and GalR3 mRNA are found in a very small number of superficial dorsal horn neurones and in lamina X, and both cell numbers and intensity of expression are very low in comparison to GalR1 in the same areas [38, 41], and the type(s) of neurones on which these receptors are expressed are unknown.

**Figure 1:**
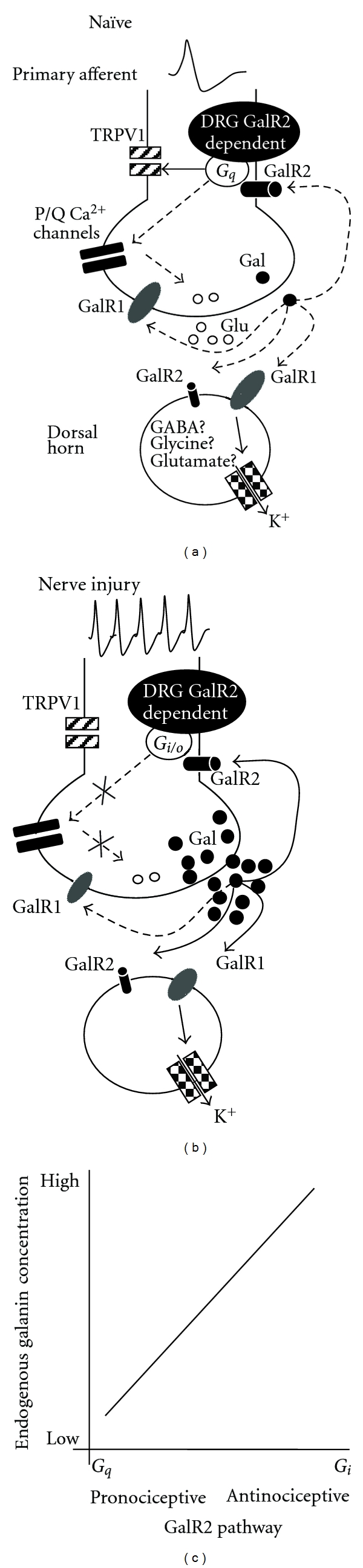
Putative galanin-mediated neuronal pro- and antinociceptive mechanisms in the dorsal horn of the spinal cord. (a) In the naïve animal, GalR1 and GalR2 are expressed on the central terminals of a large proportion of small diameter TRPV1 expressing C fibres. Galanin itself is expressed at very low levels in a small number of neurons. Peripheral activation of nociceptive C fibre afferents leads to neurotransmitter release (e.g., glutamate) at the first synapse in the superficial dorsal horn in the spinal cord, including galanin release (filled circles) [46]. In the uninjured state, galanin release is low at this synapse. Evoked galanin release or exogenous galanin is postulated to activate presynaptic GalR2 (solid arrow). This stimulates signalling through *G*_*q*_ in the central terminals, which then regulates both the sensitisation and expression of TRPV1 and hence afferent sensitivity [11, 47]. In addition, *G*_*q*_ acts on *P*/*Q* type calcium channels [11], which would serve to enhance neurotransmitter release (e.g., glutamate, open circles), enhancing excitation of postsynaptic neurons. Postsynaptic neurons express both GalR1 and GalR2. GalR1 is expressed on both excitatory (glutamatergic) and inhibitory (GABA- and glycinergic) postsynaptic neurons, and activation reduces excitability of these neurons through actions on potassium channels (checkered boxes). Postsynaptic GalR2 activation is postulated to result in the low concentration pronociceptive effects of galanin [23]. The net effect of the activation of GalR1 and GalR2 on spinal nociceptive processing will differ depending on the degree of presynaptic activation and whether excitatory or inhibitory postsynaptic neurons are affected. GalR1 is also expressed on DRG neurons, but whether presynaptic effects of galanin are also mediated through GalR1 is not yet known as there is no evidence that these receptors are functional. Dashed lines indicate minimal effects on the pathways shown. (b) After nerve injury, galanin levels are massively upregulated in DRG neurons. Up to 50% of neurons now express galanin and to a much higher level, resulting in a 120-fold increase in DRG galanin expression. There is also a small increase in galanin expression in the dorsal horn, [13] where galanin is largely found in inhibitory neurons [48, 49]. Spontaneous firing increases in primary afferents, and galanin release into the dorsal horn is increased after both nerve injury [46] and nociceptor stimulation [46]. Spinal GalR levels are only minimally altered under these conditions. Increased galanin release into the dorsal horn would increase basal activation of presynaptic GalR2, which under high galanin concentrations couples to *G*_*i*/*o*_. TRPV1 sensitisation is therefore reduced. *G*_*i*/*o*_ coupling also stops the activation of calcium channels thereby greatly reducing glutamate release and hence nociceptive input to the dorsal horn [25]. In addition, galanin exerts greater postsynaptic effects, effectively reducing central sensitisation [27, 44, 45]. In nerve injury, therefore, increased endogenous or exogenous galanin enhances these actions and results in antinociception. (c) (Inset) A schematic representation of the galanin concentration-dependent system. Pronociceptive actions are exerted by low-concentration galanin when GalR2 couples to *G*_*q*_. This then activates the protein kinase C-phospholipase C pathway to lead to enhanced nociceptor excitability and behavioural hypersensitivity. When galanin concentrations are higher, for example, after nerve injury, GalR2 couples to *G*_*i*_, reducing nociceptor excitability through inhibition of peripheral sensitisation.

Galanin has actions at both pre- and postsynaptic sites in the dorsal horn [27] (Figure 1). Galanin exerts presynaptic inhibition of neurotransmitter release, through activation of presynaptic GalR2, reducing primary afferent input into the dorsal horn [25, 42]. Activation of postsynaptic GalR1 in superficial dorsal horn laminae [38] leads to a reduction in postsynaptic neuronal excitability through activation of inward rectifying potassium currents [42]. Central sensitisation is key to the observed behavioural changes consequent to peripheral nerve injury [43], and galanin reduces central sensitisation of spinal neuronal circuits [44], particularly after nerve injury [44, 45]. There are also reports of entirely excitatory (pronociceptive) effects of galanin on wide dynamic range spinal neurons [20], possibly mediated through GalR2 [23]. Data derived from studies on intrathecal administration of the galanin fragment, Gal2-11, which is an agonist for GalR2/3, and as there is very little or no GalR3 expressed, indicates that many of the concentration-dependent effects of intrathecal galanin are exerted through GalR2, despite the relatively small numbers of spinal neurons expressing GalR2 mRNA [23]. 

 The contributions of different GalR subtypes to spinal nociceptive processing are still unclear, and, unfortunately, the development of GalR knockout (KO) animals has not greatly clarified this area [53]. GalR1 knockout animals have only subtle differences from wild types [54], possibly as a result of altered expression of GalR2 and GalR3 in these animals [54, 55]. Intact GalR2 KO animals have no observable nociceptive phenotype, and the nociceptive responses to exogenous galanin have not been investigated in these GalR2 KO animals. Pharmacological investigation had originally suggested that GalR2 may mediate the pronociceptive effects of spinal galanin and that, after nerve, injury GalR1 may underpin the antinociceptive actions [23].

An additional layer of complexity arises from the intracellular signalling of the galanin receptors, as the second messenger cascades activated give rise to different functional outcomes. All the reported galanin receptors are known to couple to *G*_*i*/*o*_ and inhibit adenylyl cyclase activity. GalR1 and GalR3 activation then results in neuronal hyperpolarization, as a result of increased potassium conductance. GalR2, however, can couple to *G*_*i*/*o*_ and *G*_*q*_ [56]; activation of the latter G protein results in activation of the phospholipase C-protein kinase C pathway [57], which appears to be essential to the GalR2 mediated pronociceptive effect [58]. Activation of *G*_*i*_ and *G*_*q*_ proteins is fundamental to nociceptive processing, resulting in anti- and pronociceptive effects, respectively, when activated through different GPCRs, in the P2Y family [59]. This suggests that different *G* protein activation by GalR2 may result in pro- or antinociceptive downstream effects. In other galanin receptor systems, opposing effects can be evoked by activation of these G-proteins by the same receptor, for example, in a model of epilepsy, GalR2-*G*_*i*_ activation is antiepileptic and GalR2-*G*_*q*_ is proepileptic [60]. In addition, different agonist concentrations have also been reported to exert opposing effects in different receptor systems, for example, low concentrations of angiotensin II lead to an inhibition of vesicular neurotransmitter release whereas high concentrations potentiate exocytosis [61]. It is hypothesised that when galanin expression rises to high levels within the peripheral nervous system, such as after peripheral nerve injury, and is released into the dorsal horn [46, 62], GalR2 activation switches from a *G*_*q*_- (low galanin concentration) to a *G*_*i*/*o*_- dependent pathway (high galanin concentration), that is from a pro- to antinociceptive signalling pathway [59].

Thus, the biphasic concentration-dependent actions of galanin on spinal nociception may occur through a combination of activation of different galanin receptors (GalR1 and GalR2), expressed on different dorsal horn neurons (inhibitory or excitatory), or acting at different sites (pre- and post-synaptic), and/or through activation of different signal transduction pathways (which are concentration-dependent GalR2 activation of either *G*_*i*/*o*_ or *G*_*q*_ second messenger pathways (Figure 1).

## 3. Direct Actions of Galanin on Primary Afferent Nociceptors

Binding and expression studies have shown that GalR1 and GalR2 are found in DRG neurons [63–65], and these receptors are functional on the central terminals of primary afferents [25, 42]. Little or no GalR3 mRNA expression is found in either the DRG or spinal cord [66, 67]. Given that proteins synthesised by DRG neurons are usually transported peripherally in addition to centrally, these observations raise the possibility that galanin may modulate the function of nociceptors by actions on their peripheral, in addition to their central terminals. Galanin expression is not limited to the nervous system but has also been identified in nonneuronal peripheral tissues such as keratinocytes, sweat glands, macrophages, and blood vessels [68]. Galanin released from such peripheral sites could therefore modulate peripheral nociceptive function, for example, keratinocytes in the skin have been demonstrated to alter primary sensory neuronal function through release of various mediators [69].

Galanin enhances the excitability of TRPV1-expressing nociceptive DRG neurones both* in vitro* [11] and *in vivo* [58]. Exogenous galanin, delivered in the periphery, has been shown to modulate the properties of primary afferent nociceptors *in vivo*, in a manner similar to that seen in the spinal cord, that is, galanin exerts both facilitatory and inhibitory effects on primary afferent nociceptors [27, 51, 70]. We have shown that the opposing actions of galanin on primary afferent nociceptors are, as in the spinal cord, concentration dependent. Low concentrations of galanin sensitise primary afferent nociceptors in naïve rodents, resulting in decreased mechanical activation thresholds and increased mechanically evoked activity, whereas higher concentrations inhibit nociceptor responses. These actions are of similar magnitude in nerve-injured animals [51]. The concentration-dependent effects of galanin on primary afferents are mediated through peripheral GalR2, as both the reduction in threshold and increase in evoked activity are seen only in afferents expressing functional GalR2 [51]. Similar concentration-dependent actions of galanin have been reported in DRG neurons *in vitro,* for example, inhibition of DRG P/Q calcium channel activity through activation of GalR2 shows similar concentration dependence [11, 71]. These findings therefore suggest that galanin receptors, specifically GalR2, in primary afferent nociceptors are possible analgesic targets *in vivo*, as nociceptor properties can be directly modulated by activation of peripheral GalR2.

## 4. Galanin, Galanin Receptors, and Actions on Primary Afferent Nociceptors Following Peripheral Nerve Injury

After a nerve injury, galanin expression is upregulated in the peripheral nervous system and galanin release is enhanced in the dorsal horn [46, 62]. The advent of knockout and transgenic animals has allowed further examination of the nociceptive role played by endogenous galanin after nerve injury. This includes using galanin-promoter-driven nerve-injury-induced galanin overexpression [72] and doxycycline-induced suppression of galanin overexpression [73]. Overexpression of galanin prevented the development of mechanical allodynia after nerve injury [51, 72], and allodynia was reversed on doxycycline administration and, importantly, reestablished on doxycycline withdrawal [73]. Study of galanin knockout mice surprisingly revealed a neurotrophic effect of galanin acting through GalR2, as both galanin and GalR2 knockout animals lost a specific subset of sensory neurones [66, 74]; therefore, those nociceptive phenotypes in these transgenic models cannot be interpreted. These findings indicate a substantial contribution of albeit overexpressed, endogenous galanin to spinal nociceptive processing in nerve injury, in that increased spinal galanin release can alleviate nerve-injury-induced allodynia. Taken together, the effects of galanin overexpression indicate that physiologically, endogenous galanin exerts antinociceptive actions under conditions in which nociceptive processing is enhanced, such as peripheral nerve injury [72, 75]. 

**Figure 2:**
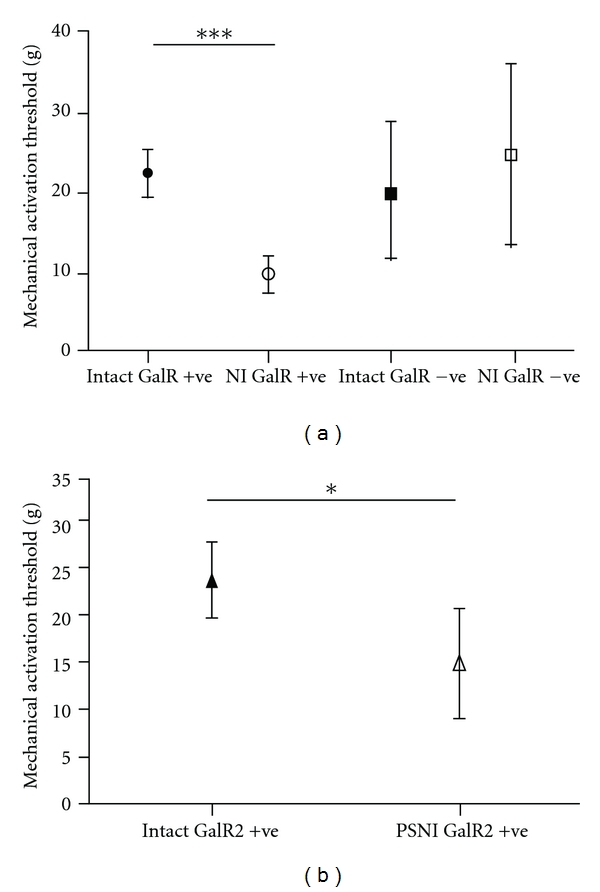
Mechanical responses of a characterised subset of C fibre nociceptor afferents expressing functional galanin receptors, in intact and PSNI-injured rats. Nociceptors were defined as those afferents with a von Frey mechanical threshold greater than 1 g [50]. Functional galanin receptors were identified in individual afferents by their response to close arterial injection of galanin and Gal2-11. Functional receptors were deemed to be present if the mechanically evoked response was increased in the afferent after galanin or Gal2-11, as at the concentrations using (close intra-arterial injection 0.1 mM) afferent responses were facilitated in both naïve and nerve injured animals [51]. (a) High threshold mechanoreceptive (nociceptive) afferents in rats with a peripheral nerve injury [14, 52] with functional galanin receptors (NI GalR+ve) had lower mechanical activation thresholds than those in uninjured rats (Intact GalR+ve). The thresholds were also lower in NI GalR+ve afferents compared to nociceptive afferents that did not express functional galanin receptors (NI and Intact GalR−ve) irrespective of whether the animals had a peripheral nerve injury or not (***P < 0.001, Kruskal Wallis test with Dunn's multiple comparison test, afferent number intact *n* = 50, PSNI *n* = 43). (b) Nociceptive afferents from animals with nerve injury with functional GalR2 (NI GalR2+ve) also had lower mechanical thresholds compared to those from naïve animals (Intact GalR2+ve). (*P < 0.05, Mann-Whitney test afferent number intact = 23, PSNI = 13).

Despite the evidence that galanin can affect the peripheral terminals of primary afferent nociceptors, most work has concentrated on the actions of galanin in the spinal cord, on presynaptic nociceptor terminals and on postsynaptic dorsal horn neurones. This is attributable to the key role of central sensitisation in altered pain behaviours consequent to nerve injury, and also because peripheral sensitisation has long been thought to contribute little to neuropathic pain. Recently, however, peripheral sensitisation has been described in nerve injury models, including reduction in primary afferent activation threshold and the onset of ongoing activity [50, 52, 76–78] and, importantly, in patients with neuropathic pain [79, 80]. When identified subsets of nociceptors are studied in inflammatory models, clear reductions in mechanical activation thresholds, that is, peripheral mechanical sensitisation, can be seen [50]. We have shown that functional GalR2 expression is a marker for those primary afferents that become sensitised to mechanical stimulation after nerve injury, that is, the afferents that express GalR2 after nerve injury are those that exhibit peripheral mechanical sensitisation (Figure 2). This is not to say that the action of galanin on primary afferents results in sensitisation and more that GalR2 is a possible target for identification and possible reversal of peripheral mechanical sensitisation. Using galanin overexpressing (GalOE) mice, we tested the hypothesis that increased endogenous galanin expression after nerve injury might directly affect the properties of the peripheral nociceptors, rather than the central processing of nociceptive inputs. In GalOE animals with peripheral nerve injury, nociceptive behaviours did not change, reduction in primary afferent nociceptor threshold was not seen, and nociceptor ongoing activity did not develop, although all of these changes were seen in wild type controls [51]. Our findings, therefore, indicate that GalR expressing primary afferent nociceptors represent at least a proportion of the population of peripheral neurons that show peripheral sensitisation after nerve injury and that increased endogenous galanin can prevent the development of peripheral sensitisation.

 In order for GalR2 in primary afferent nociceptors to represent an effective analgesic target, activation of the receptor must also be effective in chronic pain states, which is suggested by the results described above. The data in the naïve animal show that galanin can have facilitatory, in addition to inhibitory actions on primary afferents, depending on the concentration [27, 51, 70]. Facilitation would, of course, be detrimental in a chronic pain state. As described, the galanin system is highly plastic following peripheral nerve injury [26, 29], and, under these conditions, the inhibitory actions of both spinal [31] and peripheral galanin appear enhanced [27]. Determination of the contributions of specific GalR to peripheral sensitisation in neuropathy will be fundamental to the development of a potential peripheral GalR-targeted analgesic. Little, however, is known about GalR regulation in peripheral nociceptor terminals after nerve injury, due to the lack of specific GalR antibodies. GalR1 and GalR2 mRNAs are downregulated in DRG sensory neurons after peripheral nerve axotomy [66, 81], although the decrease in GalR2 is less profound [82].

Functionally, behavioural studies in GalR knockout mice do not give specific information on receptor function in peripheral nociceptors. Using methods that specifically study peripheral afferents, we have shown that peripheral activation of GalR2 modulates C fibre nociceptor function in nerve injured rats and shows a concentration dependence that is similar to that seen in naïve animals [51]. This is in contrast to the previously reported enhancement of the antinociceptive effect of galanin at the spinal level in neuropathic pain [27, 29]. While these findings might suggest that peripheral GalR may not represent good analgesic targets, we suggest that in pathological conditions when endogenous galanin levels would be dramatically increased, further GalR activation would be hypothesised to be more likely to drive GalR-*G*_*i*/*o*_- mediated signalling and would therefore result in anti-nociceptive actions.

This paper highlights peripheral GalR2 as a potential peripheral analgesic target. GalR2 activation by high concentration galanin inhibits primary afferent nociceptor activity and thereby reduces nociceptive input to the spinal cord. Once specific GalR2 pharmacological tools with favourable characteristics such as long *in vivo* half-lives are developed, peripheral antinociceptive GalR2-mediated mechanisms can be fully characterized. GalR2 may represent a therapeutic target that may be effective for the alleviation of neuropathic pain.
